# The vestibular implant: frequency-dependency of the electrically evoked vestibulo-ocular reflex in humans

**DOI:** 10.3389/fnsys.2014.00255

**Published:** 2015-01-20

**Authors:** Raymond van de Berg, Nils Guinand, T. A. Khoa Nguyen, Maurizio Ranieri, Samuel Cavuscens, Jean-Philippe Guyot, Robert Stokroos, Herman Kingma, Angelica Perez-Fornos

**Affiliations:** ^1^Division of Balance Disorders, Department of Otorhinolaryngology and Head and Neck Surgery, Faculty of Health Medicine and Life Sciences, School for Mental Health and Neuroscience, Maastricht University Medical CenterMaastricht, Netherlands; ^2^Faculty of Physics, Tomsk State UniversityTomsk, Russia; ^3^Service of Otorhinolaryngology and Head and Neck Surgery, Department of Clinical Neurosciences, Geneva University HospitalsGeneva, Switzerland; ^4^Translational Neural Engineering Lab, Center for Neuroprosthetics, Interfaculty Institute of Bioengineering, École Polytechnique Fédérale de LausanneLausanne, Switzerland

**Keywords:** vestibular implant, vestibular prosthesis, neural prosthesis, bilateral vestibular areflexia, bilateral vestibulopathy, vestibulo-ocular reflex

## Abstract

The vestibulo-ocular reflex (VOR) shows frequency-dependent behavior. This study investigated whether the characteristics of the electrically evoked VOR (eVOR) elicited by a vestibular implant, showed the same frequency-dependency. Twelve vestibular electrodes implanted in seven patients with bilateral vestibular hypofunction (BVH) were tested. Stimuli consisted of amplitude-modulated electrical stimulation with a sinusoidal profile at frequencies of 0.5, 1, and 2 Hz. The main characteristics of the eVOR were evaluated and compared to the “natural” VOR characteristics measured in a group of age-matched healthy volunteers who were subjected to horizontal whole body rotations with equivalent sinusoidal velocity profiles at the same frequencies. A strong and significant effect of frequency was observed in the total peak eye velocity of the eVOR. This effect was similar to that observed in the “natural” VOR. Other characteristics of the (e)VOR (angle, habituation-index, and asymmetry) showed no significant frequency-dependent effect. In conclusion, this study demonstrates that, at least at the specific (limited) frequency range tested, responses elicited by a vestibular implant closely mimic the frequency-dependency of the “normal” vestibular system.

## Introduction

Bilateral vestibular hypofunction (BVH) is most often a chronic condition in which patients can suffer from blurred vision (oscillopsia), impaired spatial orientation and postural instability (Brandt et al., 2010; van de Berg et al., 2011; Hain et al., 2013). These and other symptoms lead to an important decrease in physical activity, social functioning and vitality that dramatically impact the patients' quality of life (Guinand et al., 2012; Ward et al., 2013). The prognosis of BVH is poor and more than 80% of the patients do not improve (Zingler et al., 2008; McCall and Yates, 2011). Until now, treatment options are limited and with low yield (Porciuncula et al., 2012).

A vestibular implant, in a concept analogous to that of the cochlear implant, has been postulated as a possible therapeutic alternative. This idea is currently investigated by research groups in Europe and the United States. Research both from animal and human studies have demonstrated that electrical stimulation is an effective means to activate the vestibular system (Fridman et al., 2010; Guyot et al., 2011; Lewis et al., 2013; Golub et al., 2014). Considerable research efforts have been devoted to the investigation of the electrically evoked vestibulo-ocular reflex (eVOR). Promisingly, results showed that it is possible to elicit a VOR which corresponds to the plane of the canal which is innervated by the electrically stimulated nerve branch (Gong and Merfeld, 2002; Della Santina et al., 2007; Merfeld et al., 2007; Wall et al., 2007; Fridman et al., 2010; van de Berg et al., 2011; Perez Fornos et al., 2014). Current efforts focus on optimizing stimulation paradigms (Davidovics et al., 2011, 2012), on improving the alignment of the eVOR (Migliaccio et al., 2011; Dai et al., 2013; Davidovics et al., 2013), and on investigating the adaptive properties of the eVOR (Merfeld et al., 2006, 2007; Lewis et al., 2010; Guyot et al., 2011; Dai et al., 2013). Important efforts are also undertaken to improve surgical techniques (Feigl et al., 2009; Dai et al., 2011a; Bierer et al., 2012; Rubinstein et al., 2012; van de Berg et al., 2012) and to solve biomechanical issues (Wall et al., 2003; Hayden et al., 2011; van de Berg et al., 2011; Fridman and Della Santina, 2013).

The Geneva-Maastricht group has recently demonstrated that it is possible to restore the VOR in patients with BVH, using a chronically implanted vestibular implant prototype (Perez Fornos et al., 2014). During these experiments, some frequency dependent effects were observed. Frequency-dependency is a well-known feature of the vestibular system. Gain of the semicircular canals (peak eye velocity/peak head velocity) is high for middle frequencies, but decreases with lower and higher frequencies, consistent with the mechanical properties of the semicircular canals (Barnes, 1993). Interestingly, these middle frequency movements are often encountered by individuals in daily life, for example during voluntary head movements and locomotor activities (Barnes, 1993; Crane and Demer, 1997). Therefore, it is important to further investigate the frequency dependent behavior of the eVOR and how it compares to the frequency dependent characteristics of the “natural” VOR in healthy subjects. This was the main objective of this study.

## Materials and methods

### Implanted patients

Between 2007 and 2013, 11 volunteers with BVH received a vestibular implant prototype consisting of a modified cochlear implant (MED-EL, Innsbruck, Austria) with extra-cochlear branches for vestibular stimulation (Guinand et al., Submitted). The devices, inclusion criteria, and surgical techniques have been described in detail previously (Perez Fornos et al., 2014; Guinand et al., Submitted). Seven of them were available for this study (age 46–68 years; mean age 61.4 years; see Table 1). Twelve electrodes at different anatomical locations were tested: four electrodes implanted in the vicinity of the superior ampullary nerve (SAN), three electrodes implanted in the vicinity of the lateral ampullary nerve (LAN) and five electrodes implanted in the vicinity of the posterior ampullary nerve (PAN).

**Table 1:** **Main characteristics of the tested patients with bilateral vestibular hypofunction**.

**Subject**	**Sex**	**Tested electrode(s)**	**Age at implantation**	**Etiology**	**Year of implantation**	**Surgical approach**
BVH1	M	SAN; LAN	67	DFNA-9	2012	Intra-labyrinthine
BVH2	F	PAN	48	Meningitis	2012	Intra-labyrinthine
BVH3	M	SAN; LAN; PAN	66	DFNA-9	2013	Intra-labyrinthine
BVH4	F	SAN; PAN	68	DFNA-9	2013	Intra-labyrinthine
BVH5	F	SAN; LAN	67	Traumatic	2013	Intra-labyrinthine
BVH6	M	PAN	46	Idiopathic	2008	Extra-labyrinthine
BVH7	M	PAN	68	Idiopathic	2007	Extra-labyrinthine

### Healthy subjects

Seven age-matched healthy volunteers with a blank history for vestibular disorders were selected for the comparison experiments. These tests involved three men and four women (age 59–69 years; mean age 62.7 years).

### Electrical stimulation

Electrical stimulation was delivered exclusively to one vestibular electrode at a time. The activation procedure has been previously described (Guyot et al., 2011; Perez Fornos et al., 2014). Briefly, the generation of bi-directional eye movements (e.g., both leftwards and rightwards for stimulation of the LAN) with unilateral electrical stimulation requires first that a “baseline stimulation” (i.e., constant amplitude) is delivered by the vestibular electrode. Then, up- and down-modulation of this “baseline stimulation” effectively results in the generation of smooth, bi-directional eye movements.

**Figure 1:**
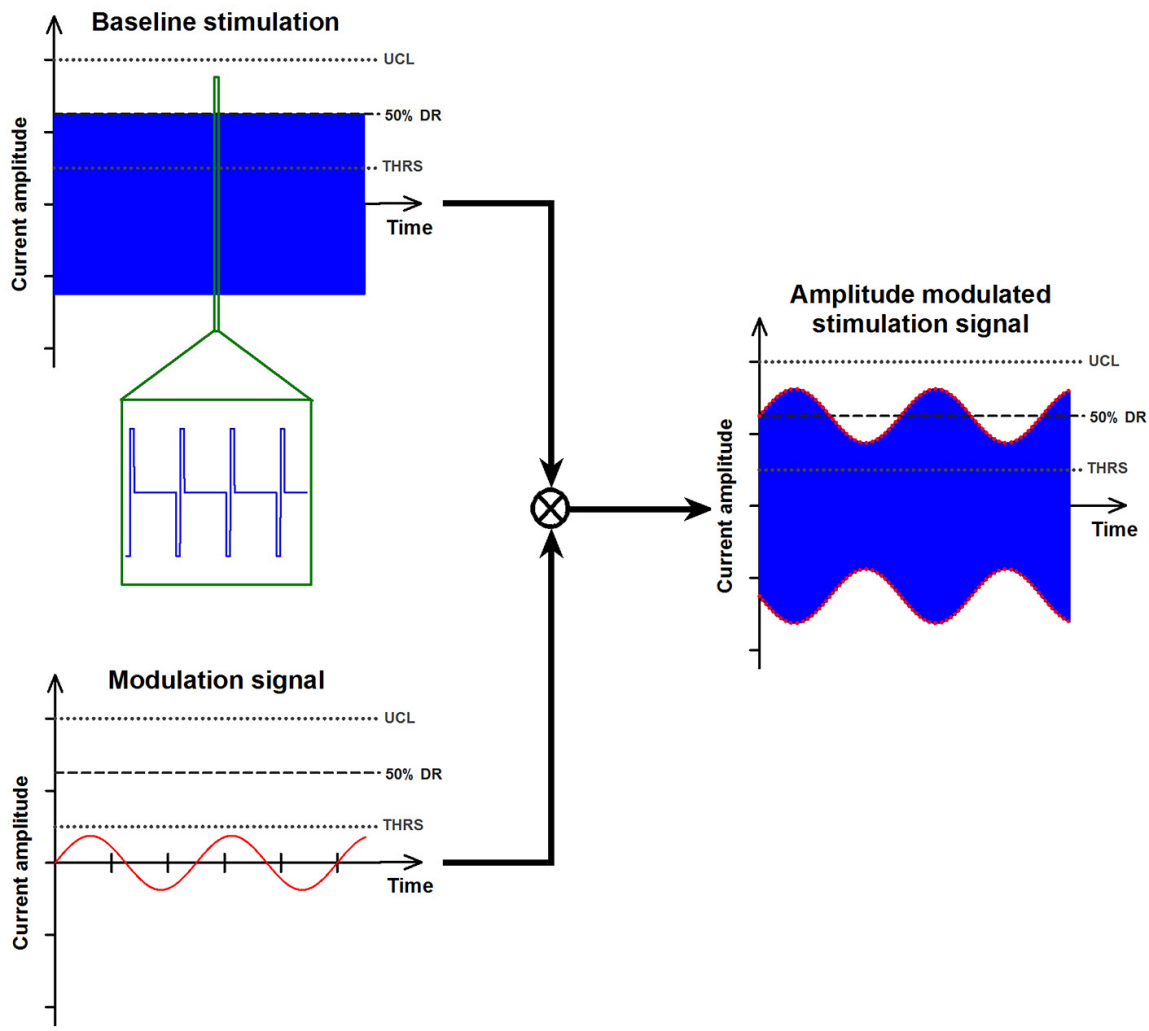
**Illustration of the electrical stimulation paradigm**. Stimulation was delivered to one vestibular electrode at a time. Patients first received baseline electrical stimulation that consisted of constant amplitude trains of biphasic, cathodic-first pulses (upper-left panel). Baseline stimulation was presented at a fixed pulse rate of 400 pulses per second. Its intensity corresponded to 50% of each electrode's previously measured dynamic range (DR; current range between the vestibular threshold -THRS- and the upper comfortable level -UCL-). Once patients were in “adapted” state, the baseline stimulation could be modulated in amplitude using a signal with a sinusoidal profile (see lower-left panel). The strength (i.e., intensity) of the modulation was kept constant and its frequency was varied between 0.5 and 2 Hz. The right panel shows an example of such an amplitude modulated stimulation signal (blue trace). The envelope of the modulation signal (red dotted lines) has been highlighted for clarity.

Stimulation consisted of amplitude modulated, charge-balanced, cathodic-first, biphasic pulses (200 μs/phase) presented at a pulse rate of 400 pulses/sec. “Baseline stimulation” amplitudes corresponded to the middle of each patient's dynamic range (see Perez Fornos et al., 2014; Guinand et al., Submitted; for details on the determination of thresholds, upper comfortable level and resulting dynamic range). Modulation strengths for each patient/electrode were selected to correspond to 50–75% of the corresponding dynamic range and were kept constant throughout the experiments. Figure 1 illustrates this electrical stimulation procedure.

### Study design

All tests were conducted in a controlled laboratory setting and performed in complete darkness. All participants (from both groups) were instructed to sit still, look in front of them and keep their eyes open during the trials. If necessary, alerting tasks were given to improve concentration and general level of arousal, in order to obtain as reproducible results as possible.

In order to test the eVOR as specifically as possible without any influences of other inputs like residual vestibular function, the eVOR-experiments were conducted in stationary conditions (e.g., without any head or body movement). Patients sat comfortably in a stationary chair while the implant was activated. Each electrode was separately tested with a fixed sequence of approximately 60 sinusoidal cycles of amplitude modulated electrical stimulation (see details in Section Electrical Stimulation). The strength (i.e., intensity) of modulation was kept constant throughout experimental trials and 3 modulation frequencies (0.5, 1, and 2 Hz) were tested. Lower modulation frequencies were intentionally excluded, since previous investigations (in exactly the same conditions) showed only very small eVOR responses at these frequencies (Perez Fornos et al., 2014). Furthermore, 60-cycle trials at low modulation frequencies below 0.5 Hz resulted in very long sessions, which severely compromised the attention of the patients for the rest of the testing session (Perez Fornos et al., 2014). All tests for a given electrode were performed on the same day.

Modulation of the frequency of the electrical stimulus would correspond in real life to modulation of the frequency of the head velocity stimulus in dynamic situations. Therefore, the eVOR obtained by electrical stimulation in patients with BVH was compared to the “natural” VOR obtained in healthy volunteers during velocity controlled whole body rotations. Healthy volunteers were subjected to 60-cycle trials of horizontal whole body rotations in a custom-made, velocity-controlled rotatory chair (Nystagliner Pro; Erich Jaeger Gmbh). Rotations followed the same sinusoidal profile as electrical stimuli (same frequency range of 0.5, 1, and 2 Hz) and had a peak velocity of 30°/s.

### Eye movement recording and analysis

**Figure 2:**
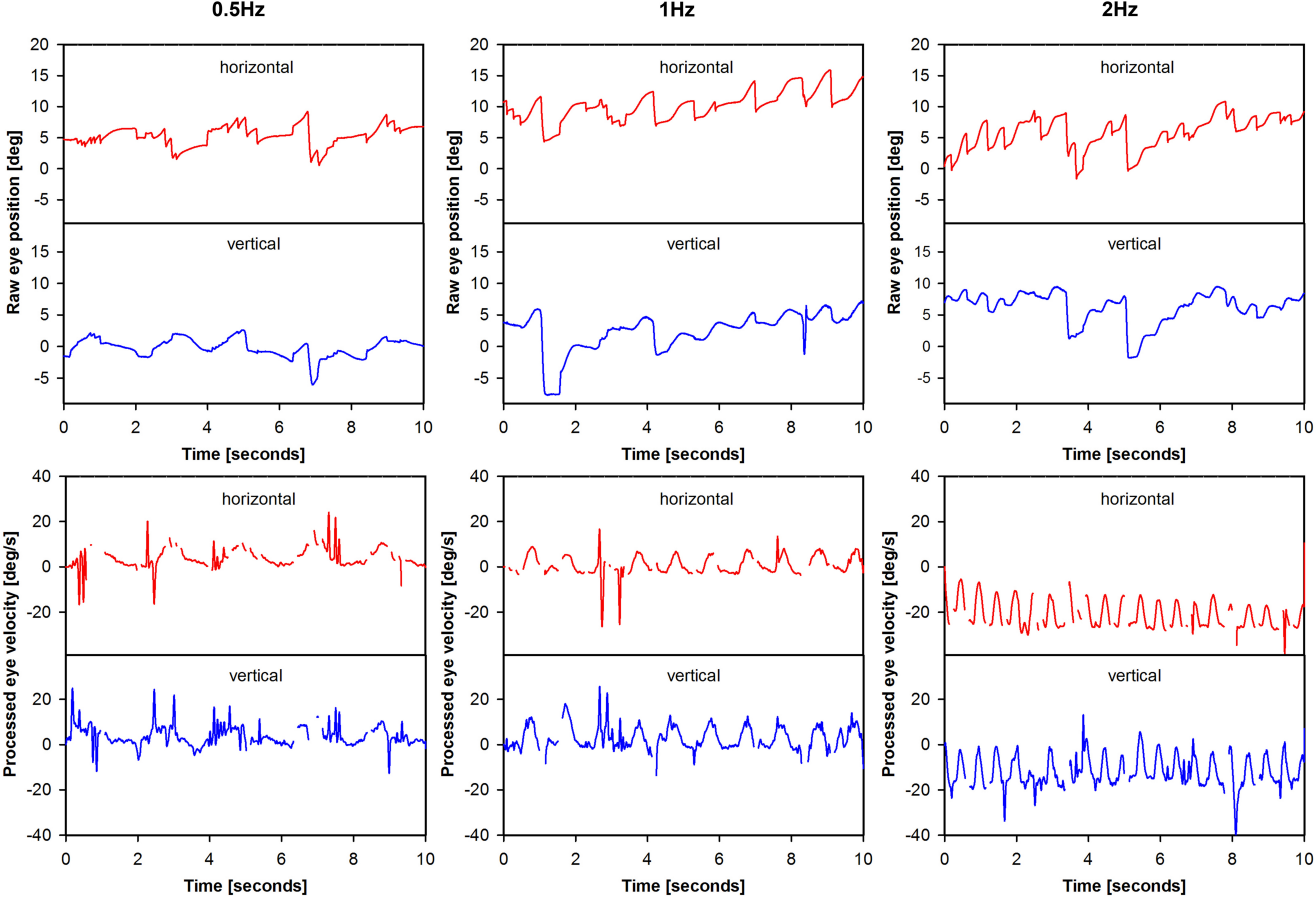
**Illustration of eye movement data processing**. The figure presents eye movement data tracings for patient BVH1-LAN. Two steps are illustrated for each modulation frequency: raw eye position (e.g., before any processing was performed) and processed eye velocity data.

Bidimensional eye movements (i.e., horizontal and vertical eye position, no torsion) were recorded with the EyeSeeCam system (EyeSeeCam VOG; Munich, Germany) (Bartl et al., 2009; Perez Fornos et al., 2014). Motivation for this choice, as well as the data-processing using cycle-by-cycle analysis and calculation of gain were described previously (Perez Fornos et al., 2014; Guinand et al., Submitted). An example of eye movement data processing is presented in Figure 2. Analysis was performed on as many valid cycles (free of saccades and blinks) as possible (minimum 43, maximum 60).

Total peak eye velocity was calculated as the square root of the sums of the squares of horizontal and vertical peak eye velocity. To facilitate the analysis of the frequency-dependent behavior of peak eye velocities of different magnitudes, peak eye velocities per modulation frequency were normalized to the highest measured peak eye velocity per electrode. Angle of the (e)VOR (with respect to the horizontal axis) was defined as the angle between the horizontal and vertical peak eye velocity (Guinand et al., Submitted). The habituation-index was determined by the mean peak eye velocities of the last 10 cycles, divided by the mean peak eye velocities of the first 10 cycles. The asymmetry-index (ratio of excitatory/inhibitory half cycle gain) of the most prominent component (horizontal or vertical) was calculated as (excitatory half cycle gain—inhibitory half cycle gain)/(excitatory half cycle gain + inhibitory half cycle gain) (Dai et al., 2011b) and converted into an absolute value.

### Statistics

Since normality tests conducted on individual results often failed the normality assumption, individual results (per subject/electrode) are presented as median values, as well as the 25th–75th percentiles. Group results conformed to normal distributions and are therefore presented as mean values ± standard error of the mean (SEM).

Statistics were performed using analysis of variance (ANOVA) modules from IBM SPSS Statistics v22 (IBM Corporation, New York, United States of America). Raw scores were used as input for comparative tests regarding angle, habituation-index and asymmetry-index. For total peak eye velocity, variance differed between the groups. Therefore, data were first normalized before proceeding to statistical analysis. A significance level of 0.05 was chosen to detect significant differences within and across groups.

### Ethical considerations

This study was in accordance with the Declaration of Helsinki (amended version 2013). The testing protocol was approved by the ethical committees of the Maastricht University Medical Center (NL36777.068.11/METC 11-2-031) and the Geneva University Hospital (NAC 11-080).

## Results

### Characteristics of the eVOR

The first objective of this paper was to describe the main characteristics of eVOR-responses. Four main characteristics were studied: total peak eye velocity, angle of the eVOR (with respect to the horizontal axis), the habituation-index over 60 cycles, and the asymmetry (ratio of excitatory/inhibitory half cycle gain) of the response.

Total peak eye velocity results obtained per electrode are presented in Table 2. Consistent with previous observations (Guinand et al., Submitted), inter-subject variability was high. The medians of the total peak eye velocity for the electrodes ranged from 0.6°/s (BVH4-PAN, 0.5 Hz) up to 21.5°/s (BVH5-SAN, 2 Hz).

**Table 2:** **Medians, 25th percentiles, and 75th percentiles of total peak eye velocity (°/s) for all electrodes, per modulation frequency (perc. = percentile)**.

**Electrode**	**Frequency (Hz)**	**Median**	**25th perc**.	**75th perc**.
BVH1-SAN	0.5	4.2	2.4	5.3
	1	5.4	3.3	7.6
	2	6.9	5.2	9.1
BVH1-LAN	0.5	6.9	5.3	8.6
	1	9.1	7.1	10.4
	2	9.3	7.3	11.4
BVH2-PAN	0.5	8.5	6.4	9.8
	1	8.3	7.8	9.0
	2	6.9	6.2	7.7
BVH3-SAN	0.5	1.1	0.9	1.5
	1	3.9	3.1	5.0
	2	5.6	4.6	7.2
BVH3-LAN	0.5	0.9	0.6	1.2
	1	1.8	1.4	2.5
	2	3.6	2.2	4.8
BVH3-PAN	0.5	2.0	1.5	2.5
	1	5.7	5.2	6.4
	2	8.6	8.0	9.7
BVH4-SAN	0.5	0.8	0.5	1.1
	1	1.4	1.0	1.7
	2	3.6	2.4	4.4
BVH4-PAN	0.5	0.6	0.5	0.8
	1	1.0	0.6	1.3
	2	1.5	1.0	2.3
BVH5-SAN	0.5	12.4	10.8	14.1
	1	13.5	10.8	17.4
	2	21.5	16.6	24.9
BVH5-LAN	0.5	6.8	6.0	8.1
	1	8.0	6.8	9.5
	2	11.1	9.0	12.9
BVH6-PAN	0.5	10.2	8.1	13.5
	1	12.5	10.6	14.7
	2	12.0	9.8	14.6
BVH7-PAN	0.5	4.0	3.5	4.4
	1	4.0	2.8	5.0
	2	5.7	4.6	7.5

**Figure 3:**
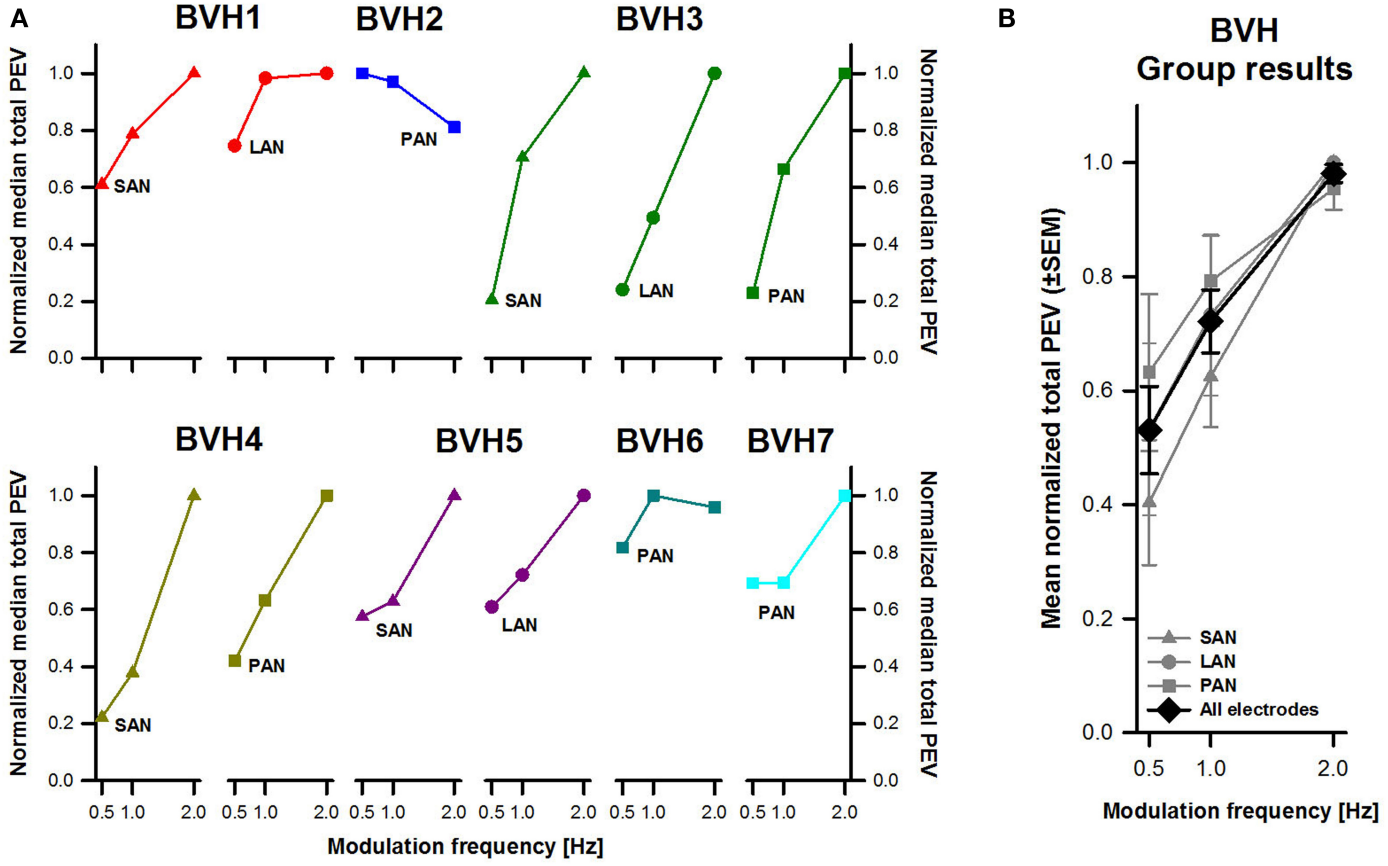
**Normalized total peak eye velocity (PEV) vs. modulation frequency**. **(A)** Each panel represents individual results obtained during stimulation for each stimulation site (SAN, LAN, PAN). **(B)** Mean results (±SEM) calculated across patients for each stimulation site (gray plots) and for all electrodes together (black plot).

To facilitate comparison of the results across patients and across frequencies, total peak eye velocity results were normalized to the highest values per electrode. Individual and pooled results revealed a clear frequency-dependent behavior for the three stimulation sites (Figure 3). In general, the lowest peak eye velocities were obtained with a modulation frequency of 0.5 Hz. Peak eye velocities progressively increased with increasing modulation frequency, reaching a maximum at 2 Hz. Note however that interestingly, patient BVH2 showed an opposite behavior (stimulation of the PAN). There was a statistically significant effect of modulation frequency [*F*_(2, 27)_ = 16.25, *p* < 0.001] but not for stimulation site. *Post-hoc* pairwise comparisons (Tukey) indicated that the difference in mean normalized peak eye velocities was statistically significant (*p* < 0.05; 0.5 Hz: 0.53 ± 0.08; 1 Hz: 0.72 ± 0.05; 2 Hz: 0.98 ± 0.02).

**Figure 4:**
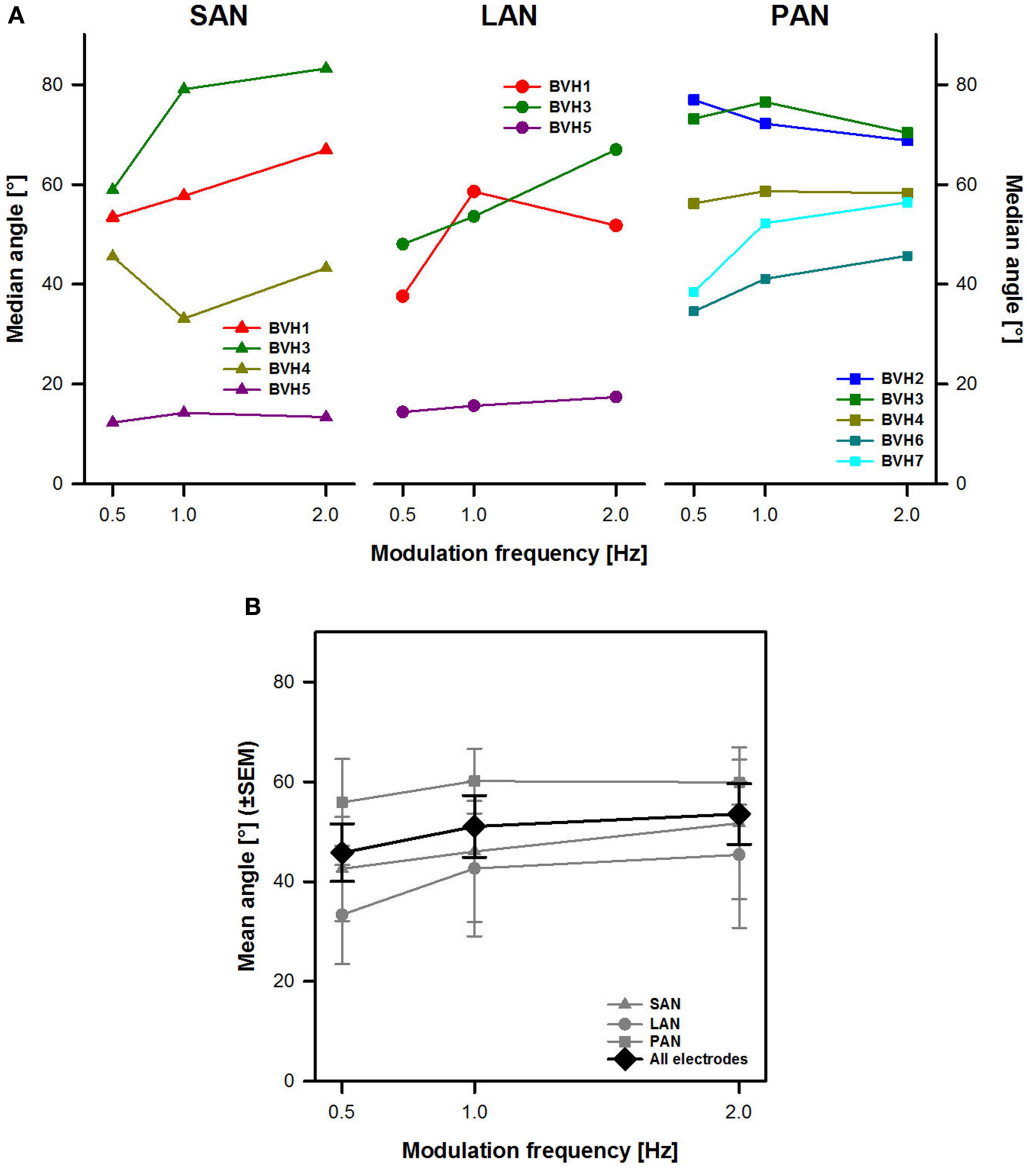
**Angle of eye movements vs. modulation frequency**. **(A)** Each panel represents individual results obtained during stimulation for each stimulation site (SAN, LAN, PAN). **(B)** Mean results (±SEM) calculated across patients for each stimulation site (gray plots) and for all electrodes together (black plot).

Figure 4A shows individual angle results (with respect to the horizontal axis) for each stimulation site. Results for each stimulation site were very variable across subjects. No clear effect of modulation frequency could be distinguished either. For example, stimulation of the SAN in BVH3 (dark green triangles in Figure 4A) showed, as expected, angles with a predominantly vertical component ranging from 59 to 83°. The eye movement response progressively shifted toward the vertical axis (the angle increased) as modulation frequency increased. However, results for the same stimulation site were completely different in the case of patient BVH5 (purple triangles in Figure 4A). Surprisingly, this patient showed median angles with a predominantly horizontal component, ranging from 12 to 14° during stimulation of the SAN. Furthermore, median angles remained relatively stable across modulation frequencies for this patient. Similar inter-subject variability was observed for stimulation of the LAN and the PAN.

Mean results across stimulation sites (gray plots in Figure 4B) showed that overall, the stimulation site with the most vertical eVOR response was the PAN (58.6 ± 5.5°). There were only very small differences in angles between stimulation of the SAN and the LAN (respectively 46.8 ± 6.1° and 40.4 ± 7.1°). Differences across modulation frequencies were small, both when each stimulation site was considered separately and when data from all stimulation sites was pooled (black plot in Figure 4B). There was no statistically significant main effect of modulation frequency or stimulation site.

**Figure 5:**
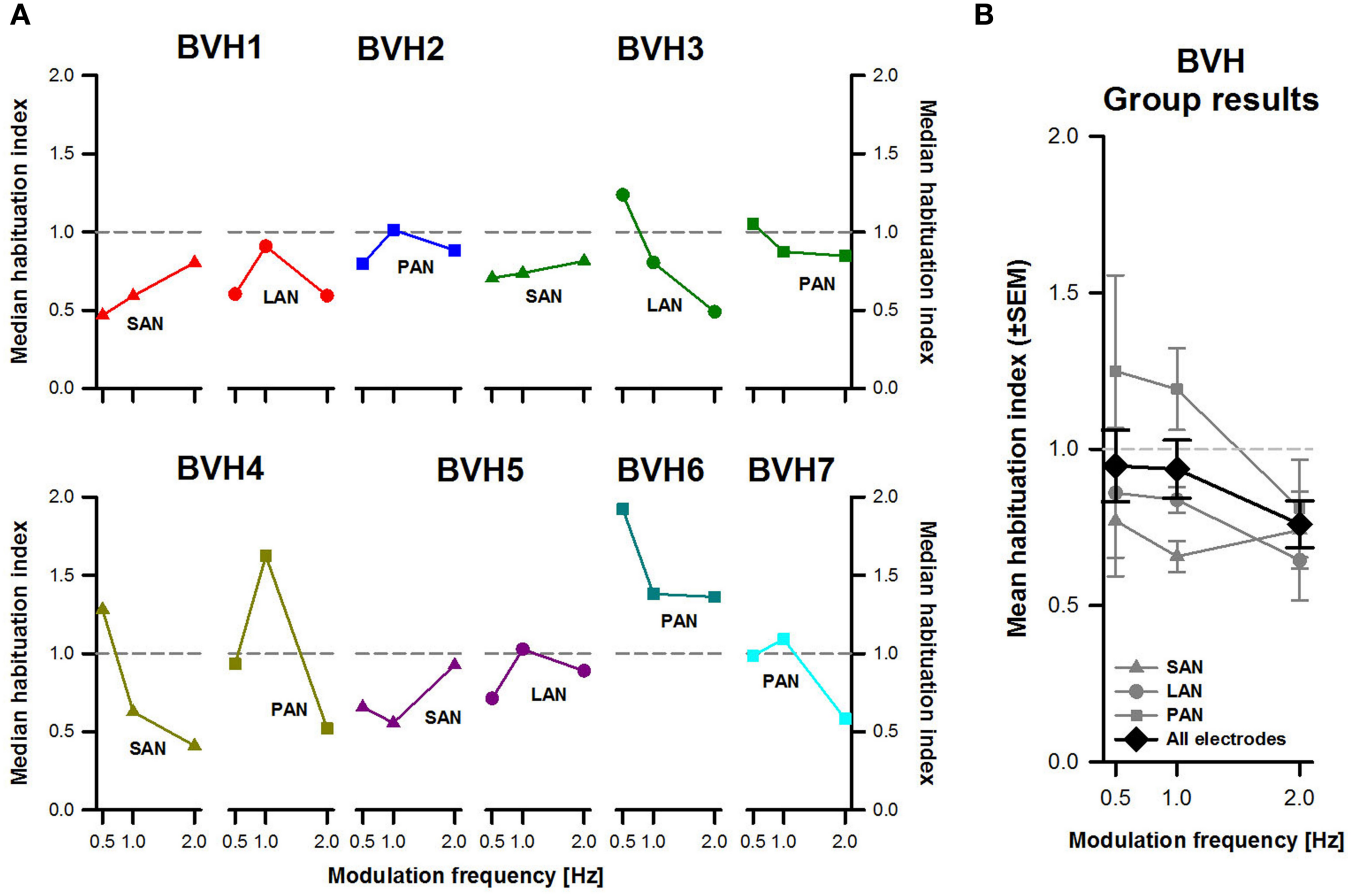
**Habituation-index vs. modulation frequency**. **(A)** Each panel represents individual results obtained during stimulation for each stimulation site (SAN, LAN, PAN). **(B)** Mean results (±SEM) calculated across patients for each stimulation site (gray plots) and for all electrodes together (black plot).

Figure 5A shows individual median habituation-indexes per patient and for each stimulation site. Results across subjects and across stimulation sites were again quite variable. Results were very variable from one stimulation site to another in the same patient (e.g., results for patient BVH3, dark green plots in Figure 5A). Habituation could also be very different when comparing the same stimulation site between patients (e.g., compare results for stimulation of the PAN, squares in Figure 5A).

No clear effect of modulation frequency could be distinguished either. While in some cases habituation seemed to be more important (i.e., indexes became lower) at higher modulation frequencies (e.g., BVH4-SAN, olive green triangles in Figure 5A), in other cases the inverse trend was observed (e.g., BVH1-SAN). Mean results across stimulation sites and for all stimulation sites together showed a clearer picture (Figure 5B). Habituation-indexes for stimulation of the PAN were in general higher (1.08 ± 0.09), reflecting less adaptation than stimulation of the SAN and the LAN (respectively 0.72 ± 0.11 and 0.78 ± 0.12). This difference was only statistically significant between stimulation of the SAN and the PAN (*p* < 0.05). Another interesting observation from pooled results was that in general, habituation was more important for the 2 Hz modulation frequency than for 0.5 and 1 Hz. However, the effect of modulation frequency, as well as the interaction effect between modulation frequency and stimulation site, were not statistically significant.

**Figure 6:**
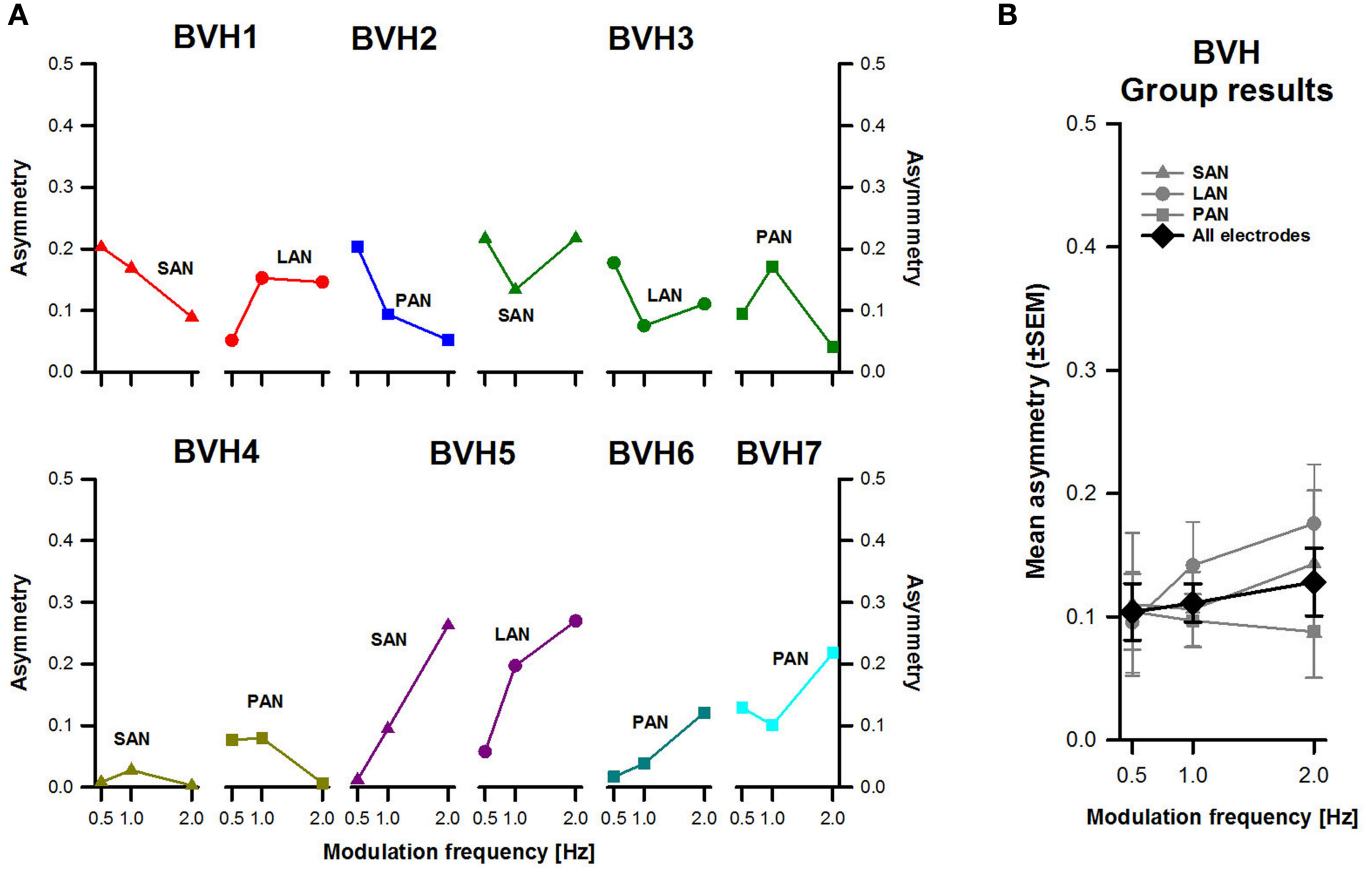
**Asymmetry-index vs. modulation frequency**. **(A)** Each panel represents individual results obtained during stimulation for each stimulation site (SAN, LAN, PAN). **(B)** Mean results (±SEM) calculated across patients for each stimulation site (gray plots) and for all electrodes together (black plot).

Figure 6 displays asymmetry-indexes for each patient and each stimulation site. Values were <0.3 in all cases. The patient showing the most asymmetrical responses was BVH5 (purple plots in Figure 6A) and the one with the most symmetrical responses was BVH4 (olive green plots in Figure 6A), particularly for stimulation of the SAN. No systematic frequency-dependent behavior was observed in individual results. Group results (Figure 6B) confirmed that asymmetry was in general low, and some variability between stimulation sites was also observed. There were no significant effects of modulation frequency or stimulation site. The interaction effect between modulation frequency and stimulation site was not statistically significant either.

### The eVOR vs. the “natural” VOR

**Figure 7:**
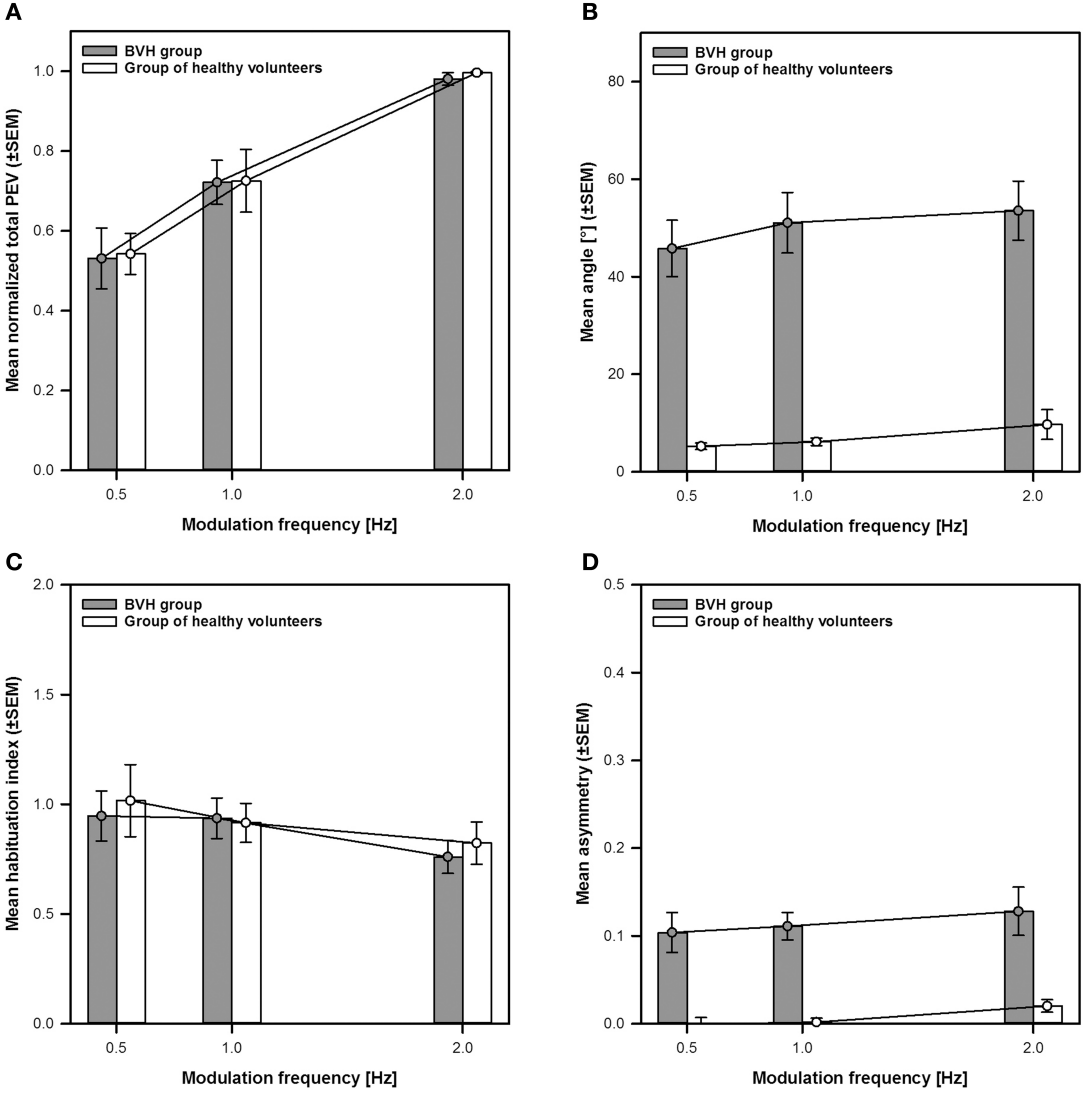
**Comparison of the main characteristics of the eVOR to the “natural” VOR**. The eVOR is represented as mean group results of the BVH-group (gray bars and circles ±SEM). The “natural” VOR is represented as mean group results of the group of healthy volunteers (white bars and circles ±SEM). **(A)** Normalized total peak eye velocity (PEV) vs. modulation frequency. **(B)** Angle of the VOR responses vs. modulation frequency. **(C)** Habituation-index vs. modulation frequency. **(D)** Asymmetry-index vs. modulation frequency.

The second goal of this study was to compare the previously described eVOR-characteristics with those of the “natural” VOR observed in the group of healthy volunteers. The results of this comparison are summarized in Figure 7.

Figure 7A compares the frequency-dependent behavior of the normalized total peak eye velocity of the eVOR to that of the “natural” VOR. From this figure it is clear that both show a strikingly similar frequency-dependent behavior, with the lower peak eye velocities measured at 0.5 Hz (0.53 ± 0.08 for the BVH-group and 0.54 ± 0.05 for the group of healthy volunteers). Peak eye velocities increase progressively with increasing frequency reaching a maximum at 2 Hz (BVH-group: 0.72 ± 0.06 at 1 Hz and 0.98 ± 0.01 at 2 Hz; Group of healthy volunteers: 0.73 ± 0.08 at 1 Hz and 0.99 ± 0.003 at 2 Hz). A Two-Way between-groups ANOVA confirmed a significant effect of frequency [*F*_(2, 51)_ = 29.39, *p* < 0.001], but no significant difference between both groups. The interaction between both variables was not significant either.

Figure 7B compares eVOR angles to those of the “natural” VOR. The angles of the “natural” VOR are close to zero (i.e., practically horizontal) and remained relatively stable across modulation frequencies, consistent with the direction of applied whole body rotations. As described previously, mean eVOR angles were much higher, with a predominantly vertical component and also remained relatively stable across modulation frequencies. A Two-Way between-groups ANOVA confirmed a significant difference between both groups [*F*_(1, 51)_ = 84.91, *p* < 0.001], but no significant effect of frequency. The interaction effect was not significant either.

Figure 7C compares the habituation-index of the eVOR to that of the “natural” VOR. Habituation-indexes were close to one (i.e., meaning very little adaptation) for both groups at 0.5 and 1 Hz and only slightly decreased at 2 Hz. There was no significant difference between groups or across modulation frequencies. The interaction effect was not significant either.

Finally, Figure 7D compares the asymmetry-indexes of the eVOR to those of the “natural” VOR. Mean asymmetry for the eVOR ranged between 0.10 (0.5 Hz) and 0.13 (2 Hz). Mean asymmetry for the “natural” VOR was much lower and close to 0, although values slightly increased at 2 Hz. A Two-Way between-groups ANOVA confirmed a significant difference between both groups [*F*_(1, 51)_ = 37.64, *p* < 0.001], but no significant effect of modulation frequency. The interaction effect was not significant either.

## Discussion

The goal of this study was to investigate how the characteristics of the eVOR change as a function of modulation frequency in the first group of patients implanted with a vestibular implant prototype, and to compare these results to the “natural” VOR responses obtained in healthy age-matched volunteers.

These results demonstrate that at least in this specific (limited) frequency range, the vestibular implant closely mimics the natural frequency-dependency of the vestibular system. Frequency showed a significant effect on the total peak eye velocity: total peak eye velocity increased with increasing frequency for both groups, without any significant effect between the groups. No significant frequency-dependent changes were observed in angle, habituation-index or asymmetry. This behavior was similar in the eVOR and in the “natural” VOR.

The increase of peak eye velocity with frequency has already been well documented in normal subjects (Barnes, 1993), but it had never been systematically evaluated in human patients with a vestibular implant. It is reasonable to hypothesize that this effect probably reflects the properties of vestibular afferents, which are the main target of electrical stimulation by a vestibular implant (Goldberg et al., 1984; Kim et al., 2011). However, it cannot be excluded that a small residual population of hair cells and more central connections can contribute to this effect (Aw et al., 2008).

The eVOR angle (with respect to the horizontal axis) was very variable across the BVH-group for the whole tested frequency range. The resulting misalignment has already been described in animals as well as humans and is attributed mainly to current spread or imprecise electrode placement (Fridman et al., 2010; Lewis et al., 2010, 2013; Dai et al., 2011c, 2013; Davidovics et al., 2011; van de Berg et al., 2011; Guinand et al., Submitted). Current spread is particularly relevant in the case of the LAN and the SAN (Figure 4A) because of their close anatomical position relative to each other (van de Berg et al., 2012). Many strategies to minimize misalignment have been investigated, such as different stimulus waveforms, precompensation (vector summation), current steering and improving electrode design, but none of these seem totally infallible (Fridman et al., 2010; Dai et al., 2011c, 2013; Davidovics et al., 2013). Fortunately, chronic stimulation experiments in animals have shown that the brain is very adaptive: it is able to significantly improve eVOR alignment, making it possible to develop an ocular response which is aligned with the axis of head motion, even when stimulating the nerve branch of a canal that is orthogonal to the axis of motion. This phenomon is called “cross-axis adaptation.” (Lewis et al., 2003, 2010, 2013; Della Santina et al., 2007; Dai et al., 2011c; van de Berg et al., 2011; Guinand et al., Submitted). Therefore, taking the adaptability of the brain into account, it should still be determined to which extent complex stimulation strategies to improve eVOR alignment will have to be incorporated into a device suited for human clinical use.

Repeated exposure to the same sinusoidal stimulus can cause a long-lasting decrease in VOR gain in animals and humans. This habituation can be frequency-specific (Dow and Anastasio, 1999). While significant habituation has been observed for low-frequency stimuli (Buettner et al., 1981; Jäger and Henn, 1981a,b; Dow and Anastasio, 1997, 1999; Clément et al., 2002), repeated stimulation at higher modulation frequencies shows little or no change in VOR gain (Ito et al., 1974; Jäger and Henn, 1981a; Dow and Anastasio, 1999). Consequently, some authors suggest sinusoidal oscillations should be limited to a few cycles or having a delay between two series of tests (Clément et al., 2002). Other key factors involved in the result of vestibular tests are general level of arousal and instruction set (Wall and Furman, 1989; Weissman et al., 1989; Barnes, 1993; Zee and Leigh, 2006). For example, it is well known and documented that results can be compromised during long testing trials. In this study, low modulation frequencies that would result in long testing times were excluded. Sixty-cycle trials were used and a delay between tests was obeyed. In these testing conditions, no significant habituation was observed. It is therefore reasonable to assume that habituation does not constitute a limiting factor in the tested frequency range.

No significant frequency-dependent changes in asymmetry were observed. However, the BVH-group showed significantly more asymmetry than the group of healthy volunteers. This could be expected, since acute unilateral vestibular stimulation (BVH-group) was compared to bilateral vestibular stimulation (group of healthy volunteers). However, it is interesting to note that the asymmetry index in the BVH-group remained relatively low (maximum 0.27) compared with previous data in unilaterally implanted monkeys (Dai et al., 2013; Guinand et al., Submitted). The major difference between both studies was the level of baseline stimulation used. Baseline stimulation in this study was set supranormally at 50% of the dynamic range, while the animal study used a lower baseline in order to mimic the physiology of normal rhesus monkey vestibular afferent fibers (Sadeghi et al., 2007; Dai et al., 2013). With a supranormal baseline, the decrease in excitatory response is counterbalanced by the increase in inhibitory response, which should improve the symmetry of the response. In other words, using a supranormal baseline corresponding to 50% of the total dynamic range, allows an equal range of stimulation currents to code head movements toward the implanted side and toward the unimplanted side. Consequently, head compensation in all directions should be enhanced (Davidovics et al., 2012). At this point it is important to point out that it is still not clear yet whether asymmetry will be an issue of clinical relevance for vestibular implants. Results on patients with a unilateral vestibular loss show that response asymmetry is generally well compensated (Curthoys and Halmagyi, 1995; Black et al., 1996; van de Berg et al., 2011). It is therefore reasonable to hypothesize that even an asymmetric eVOR might be enough to restore useful vestibular function.

Knowing the minor frequency effects on angle, habituation and asymmetry could open doors for future research. It allows these eVOR-parameters to be determined at only specific frequencies, without the need for testing the whole frequency range. This is likely to result in more precise measurements, since (1) some modulation frequencies which have specific drawbacks (i.e., low gain for low-frequencies or challenging head stabilization during rotatory tests at higher-frequencies) could be left out of the analysis and (2) time is saved in the already long testing sessions, resulting in improved patients' concentration which has to be optimal for all tests.

The fact that a vestibular implant can closely mimick the “natural” frequency-dependency of the vestibular system, is also a promising finding for device development. Since the VOR is appropriately compensated in a frequency range which is important for every-day activities (Crane and Demer, 1997), there might be no need of implementing complex stimulus processing strategies that consider frequency-dependent characteristics.

### Additional considerations

In this study, the threshold for vestibular activation was the current where the first vestibular symptom was reported or observed (Guinand et al., Submitted). This could be a change in nystagmus slow peak eye velocity or a clearly vestibular related sensation that could be below the threshold of activation of the VOR-pathway. This latter case suggests that other pathways can be activated before the VOR-pathway. These sub-VOR-threshold perceptions deserve to be investigated more in the future (Guinand et al., Submitted).

Some healthy volunteers were unable to adequately stabilize their heads at 2 Hz. This resulted in an increase in the vertical peak eye velocity component, which is in accordance with clinical experience that effectively stabilizing the head above a certain rotation frequency becomes challenging. A bite bar could be added to improve head stabilization. However, it was decided not to use a bite bar since we considered that this would impose an additional unnecessary burden to subjects. Furthermore, adding a bite bar would hinder communication with subjects. Both these considerations are particularly relevant for our long, repeated testing sessions performed in complete darkness.

Finally, a potential caveat of this study is that results are based on a small number of subjects. This suggests that results of statistical tests should be interpreted with caution. Nevertheless, the trends reported were similar across subjects and inter-subject variability was smaller than the observed effects. In these conditions, adding more subjects to the study would certainly give more statistical power to the results, but it would not fundamentally change the observed trends.

## Conclusion

A strong and significant frequency-dependency effect in total peak eye velocity was observed in the tested frequency range (0.5–2 Hz). This effect was comparable to the one observed for the “natural” VOR. (e)VOR-angle, habituation-index and asymmetry showed no significant frequency-dependent effect in any group. This study demonstrates that, at least in the specific (limited) frequency range tested, the vestibular implant closely mimics the natural frequency-dependency of the vestibular system.

### Conflict of interest statement

The authors declare that the research was conducted in the absence of any commercial or financial relationships that could be construed as a potential conflict of interest.
